# CB_1_ Blockade Potentiates Down-Regulation of Lipogenic Gene Expression in Perirenal Adipose Tissue in High Carbohydrate Diet-Induced Obesity

**DOI:** 10.1371/journal.pone.0090016

**Published:** 2014-02-25

**Authors:** Margarita Vida, Patricia Rivera, Ana Luisa Gavito, Juan Suárez, Francisco Javier Pavón, Sergio Arrabal, Miguel Romero-Cuevas, Dolores Bautista, Ana Martínez, Fernando Rodríguez de Fonseca, Antonia Serrano, Elena Baixeras

**Affiliations:** 1 Unidad de Gestión Clínica de Salud Mental, Hospital Regional Universitario de Málaga, Universidad de Málaga, Málaga, Spain; 2 Unidad de Gestión Clínica de Medicina Interna, Hospital Regional Universitario de Málaga, Universidad de Málaga, Málaga, Spain; 3 Unidad de Gestión Clínica de Anatomía Patológica, Hospital Regional Universitario de Málaga, Universidad de Málaga, Málaga, Spain; 4 Instituto de Investigación Biomédica de Málaga (IBIMA), Hospital Regional Universitario de Málaga, Universidad de Málaga, Málaga, Spain; 5 Instituto de Química Médica Lora Tamayo, Consejo Superior de Investigaciones Científicas. Madrid, Spain; 6 CIBER de Fisiopatología de la Obesidad y Nutrición (CIBEROBN), Santiago de Compostela, Spain; University of Barcelona, Faculty of Biology, Spain

## Abstract

*De novo* lipogenesis and hypercaloric diets are thought to contribute to increased fat mass, particularly in abdominal fat depots. CB_1_ is highly expressed in adipose tissue, and CB_1_-mediated signalling is associated with stimulation of lipogenesis and diet-induced obesity, though its contribution to increasing fat deposition in adipose tissue is controversial. Lipogenesis is regulated by transcription factors such as liver X receptor (LXR), sterol-response element binding protein (SREBP) and carbohydrate-responsive-element-binding protein (ChREBP). We evaluated the role of CB_1_ in the gene expression of these factors and their target genes in relation to lipogenesis in the perirenal adipose tissue (PrAT) of rats fed a high-carbohydrate diet (HCHD) or a high-fat diet (HFD). Both obesity models showed an up-regulated gene expression of *CB_1_* and *Lxrα* in this adipose pad. The *Srebf-1* and *ChREBP* gene expressions were down-regulated in HFD but not in HCHD. The expression of their target genes encoding for lipogenic enzymes showed a decrease in diet-induced obesity and was particularly dramatic in HFD. In HCHD, CB_1_ blockade by AM251 reduced the *Srebf-1* and *ChREBP* expression and totally abrogated the remnant gene expression of their target lipogenic enzymes. The phosphorylated form of the extracellular signal-regulated kinase (ERK-p), which participates in the CB_1_-mediated signalling pathway, was markedly present in the PrAT of obese rats. ERK-p was drastically repressed by AM251 indicating that CB_1_ is actually functional in PrAT of obese animals, though its activation loses the ability to stimulate lipogenesis in PrAT of obese rats. Even so, the remnant expression levels of lipogenic transcription factors found in HCHD-fed rats are still dependent on CB_1_ activity. Hence, in HCHD-induced obesity, CB_1_ blockade may help to further potentiate the reduction of lipogenesis in PrAT by means of inducing down-regulation of the *ChREBP* and *Srebf-1* gene expression, and consequently in the expression of lipogenic enzymes.

## Introduction

Over the last two decades many studies have shown that the health risks related with obesity are particularly associated with the enlarged fat depots that closely surround the viscera [1]. Obesogenic diets provoke increased fat storage of white adipose tissue, mainly in mesenteric (visceral), retroperitoneal (including perirenal) and perigonadal fat pads [2]. In humans, carbohydrate-rich diets have the most harmful effect in terms of the increase in visceral adipose tissue size. Consequently, low-carbohydrate diets appear more effective at reducing visceral fat than low-fat diets [3], [4].

Dietary carbohydrate is transformed into fat through *de novo* lipogenesis [5]. An increase in *de novo* lipogenesis appears to be an important contributor to enlarged fat mass [5]. The roles of the transcription factors liver X receptor (LXR), sterol-response element binding protein (SREBP) and carbohydrate-responsive-element-binding protein (ChREBP) are well established in the regulation of lipogenic gene expression [6]. The LXR transcription factor is expressed and activated by endogenous ligands. Activation of LXR in turn stimulates transcription of the SREBP-1 and CHREBP encoding genes (*Srebf-1* and *ChREBP*) [5], [6]. The enzymes involved in *de novo* fatty acid synthesis are acetyl-CoA carboxylase (ACC), fatty acid synthase (FAS), and stearoyl-CoA desaturase 1 (SCD1). The transcription factors LXR, SREBP and ChREBP play an important role in the regulation of the expression of the genes encoding for these three enzymes FAS, ACC and SCD1 (*Fasn*, *Acaca*, *Scd1*), leading to triglyceride storage in hepatocytes and adipocytes [5]. An increase in mRNA levels of *Srebf-1*, *Fasn* and *Acaca*, and *de novo* lipogenesis has been described in the liver and adipose tissue of animals and humans with high fat diet-induced obesity or after a carbohydrate overfeeding [7], [8]. However, lower adipose tissue levels of *Srebf-1*, *Fasn* and *Acaca* mRNA were also reported in obese compared to lean subjects [9], [10].

Besides its capacity to store and release energy when needed, the adipose tissue is also considered an endocrine organ secreting adipokines (leptin and adiponectin), endocannabinoids (anandamide, 2-arachidonoylglycerol), and pro-inflammatory cytokines (e.g., TNFα, IL-6, IL-8) that act in concert to regulate food intake and energy balance, mainly through their actions in specific brain areas [11], [12]. Much of the evidence showing the association of obesity with adipose inflammation comes from the study of visceral fat depots, including omental and mesenteric, representing a risk factor for development of the metabolic syndrome and insulin resistance [13]–[15]. Also, growing evidence supports that perivascular adipose tissues as perirenal and pericardial fat pads contribute to exacerbate metabolic syndrome [16]. Even so, the contribution of each fat depot to the pathophysiology of complicated obesity is not completely understood. In this regard, the involvement of endocannabinoids in the development of metabolic complications associated with obesity deserves particular attention [17], [18]. Experimental data suggest that the endocannabinoid system is hyper-activated in human abdominal obesity [19]. Endocannabinoids increase appetite and food intake through the activation of the highly expressed CB_1_ receptor in the mesolimbic and hypothalamic areas of the brain [20], [21]. Moreover, CB_1_ is also widely distributed throughout the body, especially in the adipose tissue, muscle and liver [22], [23]. All these organs constitute peripheral targets for the action of endocannabinoids where they are involved in the regulation of lipid and carbohydrate metabolism [23]–[26]. CB_1_ activation is associated with stimulation of lipogenesis and diet-induced obesity [17], [19]. Excess levels of endocannabinoids exert a negative impact on the control of food intake, insulin and leptin resistance, glucose tolerance, dyslipidemia, hypertension, and cardiovascular disorders. Administration of CB_1_ agonists to lean animals increases the hepatic levels of SREBP and stimulates transcription of *Fasn* and *Acaca* enzymes and *de novo* lipogenesis [24]. In contrast, the administration of CB_1_ antagonists like rimonabant inhibits lipogenesis in the liver [24], [25], [27]. Nevertheless, the influence of chronic levels of endocannabinoids in the regulation of lipogenesis in several abdominal fat depots is not fully understood. In a recent study, chronic cannabis smoking was associated with a high avidity to carbohydrate intake and with visceral adiposity [28] further supporting the notion of a close relationship between high levels of endocannabinoids, a diet rich in carbohydrate and an increase in visceral adipose tissue size.

The molecular mechanisms underlying CB_1_-mediated signalling and regulating lipogenesis are not completely known. The stimulation of lipogenesis by CB_1_ probably lies in the coupling of CB_1_ to Gi/o proteins. The stimulation of Gi/o proteins blocks the activity of adenylate cyclase (AC), protein kinase A (PKA) and adenosine monophosphate-activated protein kinase (AMPK) [29]. PKA and AMPK regulate the lipogenesis pathway negatively by inducing phosphorylation of LXR (LXR-p) and thereby down-regulating *Srebf-1* and *ChREBP* and their target genes [27], [30]–[32]. Besides inhibiting their transcription, PKA and AMPK directly phosphorylate and inactivate SREBP and ChREBP [6], [33], [34]. CB_1_ agonists stimulate the lipogenesis pathway by inhibiting PKA/AMPK through coupling the Gi/o protein [27]. Also, the CB_1_ -activated Gi/o proteins stimulate mitogen activated protein kinase (MAPK) pathways [35], including extracellular signal-regulated kinases 1/2 (ERK1/2), which have been implicated in adipogenesis [36]. Recently, the phosphorylation of SREBP by the ERK1/2 pathway has been related with fatty liver and visceral obesity [37]. Together, it seems that CB_1_ activation can stimulate lipogenesis by inhibiting the PKA/AMPK pathways while stimulating the MAPK/ERK1/2 pathways through coupling the Gi/o proteins.

In the present study we evaluated the role of CB_1_-mediated activity in perirenal fat -a part of the retroperitoneal depot white adipose tissue- of lean and diet-induced obese animals focusing on the study of: i) expression of CB_1;_ ii) expression of the transcription factors *Lxr*, *Srebf-1* and *ChREBP*, iii) expression of their lipogenic target gene enzymes *Acaca*, *Fasn* and *Scd1*, and finally, iv) the CB_1_-mediated signalling machinery involved in the expression and activity of lipogenic factors. To determine the direct involvement of CB_1_ in these events we used the CB_1_ antagonist N-(piperidin-1-yl)-5-(4-iodophenyl)-1-(2,4 dichlorophenyl-4-methyl-1H-pyrazole-3-carboxamide), also known as AM251, as a pharmacological tool [38].

## Materials and Methods

### Ethics Statement

All experimental procedures with animals were carried out in strict accordance with the recommendations in the European Communities directive 2010/63/EU and Spanish legislation (Real Decreto 53/2013, BOE 34/11370–11421, 2013) regulating the care and use of laboratory animals. The protocol was approved by the Ethics Committee for Animal Experiments of the University of Málaga (Permit number: 2011/0001). Male Wistar rats (Charles Rivers, Barcelona, Spain) were housed in pairs in cages maintained in standardized conditions of animal facilities at 20±2°C room temperature, 40±5% relative humidity and a 12 h light/dark cycle with dawn/dusk effect. At the beginning of the experiments the rats weighed 200–250 g and were 10–12 weeks old. Water and food were available *ad libitum* throughout the course of the study. Animals were anaesthetized (sodium pentobarbital, 50 mg/kg body weight, i.p.) in a room separate from the other experimental animals and sacrificed by decapitation. All efforts were made to minimize animal suffering.

### Measurement of Body Weight Gain

Rats (n = 16 per group) were fed *ad libitum* for 12 weeks with three different types of diet: a regular chow diet (standard diet, STD) purchased from Harlam Teklad, Madison WI, a high-fat diet (HFD, 60% fat diet; D12492), or a high-carbohydrate diet (HCHD, 70% carbohydrate diet; D12450B) both purchased from Research Diets Inc. (New Brunswick, NJ, USA). The HFD and HCHD were used in order to induce obesity. The STD contains 12.12 kJ/g (6% fat, 20% protein), the HFD diet consisted of 21.90 kJ/g (of which, 20% of the metabolizable energy content was protein, 20% carbohydrates and 60% fat) and the HCHD had 16.09 kJ/g (with 20% protein, 70% carbohydrates and 10% fat). The HFD contained fat constituted by soybean oil (9.26 kJ% of total fat content) and lard (90.74 kJ% of total fat content) while the HCHD contained carbohydrates constituted by corn starch (45 kcal% of total carbohydrate content), maltodextrin (5 kJ% of total carbohydrate content) and sucrose (50 kJ% of total carbohydrate content). The body weight gain was measured every two days for 12 weeks.

### Subchronic AM251 Treatment

After 10 weeks (divergence of weight curves) half of each group of diet-fed rats (n = 8 per group) received a daily intraperitoneal (i.p.) injection of vehicle (1 mL/kg of 10% Tocrisolve in saline) or CB_1_ receptor inverse agonist AM251 (Tocris Bioscience, Bristol, UK), at 3 mg/kg in 10% Tocrisolve, over 14 days, while the diets remained unchanged. The cumulative food/calorie intake and body weight gain were measured every day during the 14 days of treatment. Thus, we generated six experimental groups: STD-vehicle and STD-AM251, HFD-vehicle and HFD-AM251, and HCHD-vehicle and HCHD-AM251.

### Sample Collection, Perirenal Adipose Tissue Extraction and Histological Examination

Free-feeding animals from the six experimental groups were anaesthetized (sodium pentobarbital, 50 mg/kg, i.p.) two hours after the last dose of treatment in a room separate from the other experimental animals. Blood samples were collected from the orbital cavity into tubes containing EDTA-2Na (1 mg/mL blood) and centrifuged (1600 g for 10 min, 4°C), and all plasma samples were frozen at −80°C for biochemical analysis. Perirenal adipose tissue (PrAT) was dissected (16 animals per diet group), immediately frozen in liquid nitrogen and kept at −80°C until mRNA or protein extraction for subsequent analysis. The PrAT collected from rats (16 animals per diet group) were also prepared for histological examination. Samples were cut into small pieces, immediately fixed in 4% paraformaldehyde for 24 hours, and embedded in paraffin. Then samples were cut by microtome (5-µm thick), mounted on D-polylysinated glass slides, deparaffinized in xylene, and stained with haematoxylin and eosin for the evaluation of adipocyte size, tissue structure and inflammatory state by using an optical microscope (Olympus).

### RNA Isolation and cDNA Synthesis

Total RNA from PrAT sections (250 mg) was extracted using Trizol reagent (Invitrogen, Carlsbad, CA) and purified with Rneasy Mini Kit (QIAGEN GmbH, Hilden, Germany) in accordance with the manufacturer’s instructions. RNA concentration and purity were determined using a Nanodrop TM spectrophotometer ND-1000 (Thermo Scientific). RNA (1 µg) was reverse transcribed with Transcriptor First Strand cDNA Synthesis kit (Roche Applied Science, Mannheim, Germany).

### Real-time Quantitative Polymerase Chain Reaction (qPCR) and Gene Expression Analysis

The expression of genes encoding for rat CB_1_ *(Cnr1),* LXRα *(Lxrα),* SREBP *(Srebf-1),* ChREBP *(ChREBP),* ACCα *(Acaca),* FAS *(Fasn)*, and SCD1 (*Scd1*) was measured by qPCR. As reference genes we used rat ribosomal protein L19 (*Rpl19*) and rat Sp1 transcription factor (*Sp1*). Specificity for each primer was tested using BLAST analyses on National Center for Biotechnology Information (NCBI) database. The gene symbol, GeneID, primer sequences, and amplicon length are listed in Table 1. Polymerase chain reactions were carried out on the CFX96 Real-Time PCR detection system (Bio-Rad, Hercules, CA) for each cDNA template and amplified in 15 µl reaction volume containing 1x SYBR Green PCR master mix (Rox; Applied Science, Mannheim, Germany, Roche), and the Forward (Fw) and Reverse (Re) primers mix. Each assay included, in duplicate, a no-template control (NTC, water) to verify no amplification of contamination. Thermocycling parameters were the same for each amplicon following the supplier’s instructions with one cycle at 95° for 10 min for initial denaturation, followed by 40 cycles of two-steps at 95° for 10 s and 60° for 30 s, ending with a melting curve. Specificity was assessed with melting curves.

**Table 1:** Primer sequences used for real-time quantitative PCR.

Gene symbol	GeneID		Primer sequence 5′ to 3′	GeneBank AN*	Amplicon length
*Cnr1*	25248	Fw	ACAGCCAGCATGCACAGGGC	NM_012784.3	94
		Re	CGGCGGACGTGTCTGTGGAC		
*Acaca*	60581	Fw	TGTGGGCTGGCTGGGGTCAT	NM_022193.1	104
		Re	CGCCCACATGGCCTGACTCG		
*Fasn*	50671	Fw	AGTTTCCGTGAGTCCATCCT	NM_017332.1	182
		Re	TCAGGTTTCAGCCCCATAGA		
*Scd1*	246074	Fw	GAAGCGAGCAACCGACAG	NM_139192.1	71
		Re	GGTGGTCGTGTAGGAACTGG		
*Lxrα*	58852	Fw	AGGGCTCCAGGAAGAGATGT	NM_031627.2	76
		Re	CAACTCCGTTGCAGAGTCAG		
*Srebf-1*	78968	Fw	CGTGGATGGGGACTGGGCTGTA	XM_001075680.2	172
		Re	CCTGTCTCCATCAGCTGCCCCT		
*ChREBP*	171078	Fw	ACAACCCCTGCCTTACACAG	FN432819.1	180
		Re	GAGGTGGCCTAGGTGGTGTA		
Reference genes					
*Rpl-19*	81767	Fw	TGCCGGAAGAACACCTTG	NM_031103.1	121
		Re	GCAGGATCCTCATCCTTCG		
*Sp1*	24790	Fw	GCTATAGCAAACACCCCAGGT	NM_012655.1	115
		Re	GATCAGGGCTGTTCTCTCCTT		

*Accesion number.

Fw = Forward.

Rev = Reverse.

Raw fluorescence data were submitted to the online Miner tool (http://www.miner.ewindup.info/) for calculation of Cq and efficiency values for each experimental set [39]. Cq values were converted into relative expression values taking into account amplification efficiencies, inter-run variations, and normalization factors by means of Biogazelle’s qbase^ PLUS^ software (Biogazelle, Zwijnaarde, Belgium) using both *Rpl19* and *Sp1* as reference genes [40]. Repeatability between replicates was accepted when the ΔCq value was <0.7. For all reference and target gene studies, two independent biological samples of each experimental condition were evaluated in technical duplicates. Finally, Calibrated Normalized Relative Quantity (CNRQ) values were exported from the qbase^PLUS^ software and investigated statistically.

### Protein Extraction and Western Blot Analysis

PrAT samples were disrupted in lysis buffer (0.2% SDS, 1 mM EDTA, 1% NaDOC, 150 mM NaCl, 1% Triton and 50 mM Tris-HCl pH 7.6) supplemented with a cocktail of protease inhibitors (Roche Complete tablets). The suspension was shaken for 30 min at room temperature and centrifuged at 13000 rpm for 45 min at 10°C, recovering the soluble fraction below the fat ring. Protein concentration was determined by Bradford protein assay. Protein extracts were diluted 1∶1 in 2X sample buffer containing DTT and boiled for 5 min before submitting to SDS-PAGE. Samples (50 µg of total proteins each) were resolved in gradient SDS-PAGE gels (Bio-Rad laboratories, Inc) and blotted onto nitrocellulose membranes (Bio-Rad). Membranes were blocked in TBS-T (50 mM Tris-HCl, pH 7.6; 200 mM NaCl, and 0.1% Tween-20) with 2% BSA. Specific proteins were detected by incubation in TBS-T, 2% BSA for 2 h with the corresponding primary antibodies: rabbit anti-ACCαβ, anti-FAS, anti-SCD1, anti-PKA, anti-PKAαβ-Thr198, anti-AMPKα, anti-AMPKα-Thr172, anti-ERK1/2, anti-ERK1/2-Thr202/Tyr204 and anti-Actin antibodies, all purchased from Cell Signaling Technology Inc. (MA, USA). Rabbit anti-LXRα was purchased from Abcam (Cambridge, UK), and Anti-Adaptin γ antibody was from BD Biosciences (NJ, USA). After extensive washing in TBS-T, anti-rabbit-HRP conjugated secondary antibody (Promega, Madison, MI, USA) was added for 1 h. Membranes were subjected to extensive washings in TBS-T and the specific protein bands were revealed using the enhanced chemiluminiscence detection system (Santa Cruz, Biotechnology Inc. CA, USA), in accordance with the manufacturer’s instructions, and images were visualized in an Autochemi-UVP Bioimaging System. Bands were quantified by densitometric analysis performed by ImageJ software (Rasband, W.S., ImageJ, U.S., National Institutes of Health, Bethesda, MA, USA, http://imagej.nih.gov/ij, 1997–2012).

### Statistical Analysis

All data for graphs and tables are expressed as the mean ± standard error of the mean (SEM). Statistical analysis of the results was performed using the computer program GraphPad Prism version 5.04 (GraphPad Software Inc., San Diego, CA, USA). The significance of the differences between groups was evaluated by one-way analysis of variance (ANOVA) followed by a *post hoc* analysis for multiple comparisons (Bonferroni’s *post hoc*) or by one-tailed Student’s *t* test with the *Welch* correction applied when appropriate (no equal variances assumed) when comparing only two conditions. A *p*-value below 0.05 was considered statistically significant (*, # *p<*0.05; **, ## *p<*0.01; ***, ### *p<*0.001).

## Results

### Effect of AM251 on Body Weight and CB_1_ Expression in PrAT of Rats Fed High-carbohydrate or High-fat Diets for a Long Time

**Figure 1:**
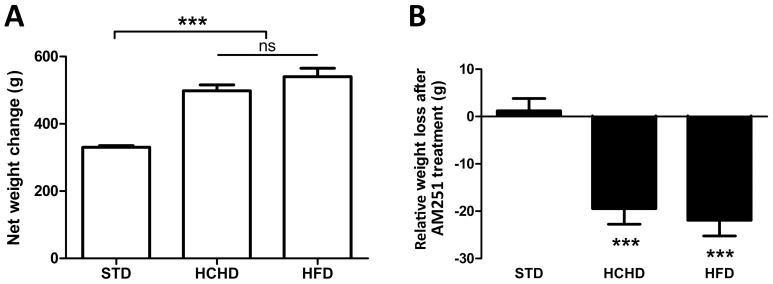
Effect of AM251 on body weight in rats fed standard or obesogenic diets. Net body weight gain was evaluated in rats fed for 10 weeks on a STD, HCHD or HFD. Columns are means ± SEM (n = 16 animals per group). Data were analyzed by one-way ANOVA and Bonferroni’s *post-hoc* test. ***p<0.001 denotes significant differences compared with the STD-fed group (**A**). Relative body weight loss was evaluated in STD, HCHD or HFD-fed animals after 14-day exposure to vehicle or AM251 (3 mg/kg, daily, i.p.). Columns are means ± SEM (n = 8 animals per group). Each pair of treatment groups was analyzed by Student’s *t* test. ***p<0.001 denotes significant differences compared with the corresponding vehicle-treated group (**B**).

As expected, rats fed a HCHD or HFD became obese and at week 10 showed a significant (*p*<0.001) weight gain over the rats with normal growth maintained on a regular chow diet (Fig. 1A). In agreement with our previous results, rats showed a steady weight gain up to a maximum body weight at week 10 of the HCHD and HFD, followed by a plateau to the end of the study [23]. As found previously, a reduction in body net weight was observed in the two groups of rats fed obesogenic diets as early as day 4 of AM251 treatment [23], [41]. Thus, compared with the corresponding groups receiving vehicle, at the end of the AM251 treatment (day 14) the body net weight was reduced up to 1.2 g ±2.5 in the STD fed rats, 19.4 g ±3.2 (p<0.001) in the HCHD-fed rats and 21.9 g ±3.2 (p<0.001) in the HFD-fed rats (Fig. 1B).

**Figure 2:**
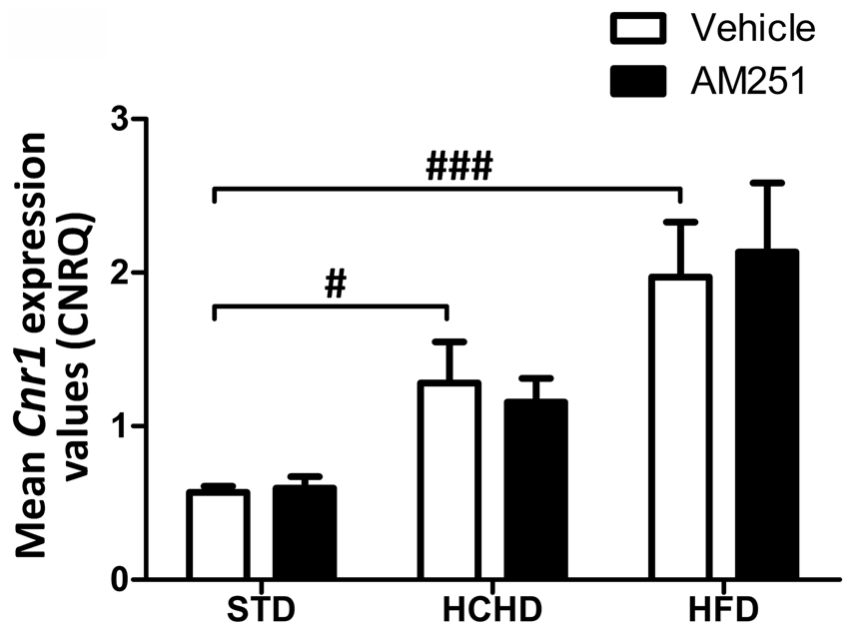
Effect of obesogenic diets and AM251 on *Cnr1* expression in PrAT. The *Cnr1* gene expression was determined by qPCR analysis in PrAT of STD, HCHD or HFD-fed animals after 14-day exposure to vehicle or AM251 (3 mg/kg, daily, i.p.). The *Cnr1* expression was normalized to the geometric mean expression of the two reference genes *Rpl19* and *Sp1.* The average calibrated normalized relative *Cnr1* gene expression values in arbitrary units (CNRQ ± SEM) is shown in the figure (n = 8 rats per group). Columns are means ± SEM. Differences between diets were analyzed by one-way ANOVA and Bonferroni’s *post-hoc* test, and comparisons between groups treated with AM251 or vehicle were analyzed by Student’s *t* test. #*p*<0.05 and ###*p*<0.001 denote significant differences compared with the STD-fed group treated with vehicle.

We examined the *Cnr1* gene expression in PrAT from rats fed a HCHD and HFD as compared with rats fed a regular chow diet and the effect of the AM251. The qPCR analysis revealed that *Cnr1* gene expression was significantly increased, by 2.25-fold in HCHD-fed rats (*p*<0.05) and 3.40-fold in HFD-fed rats (*p*<0.001) treated with vehicle compared with the STD-fed animals treated with vehicle (Fig. 2). The treatment with AM251 did not significantly alter the *Cnr1* expression levels in any of the nutritional conditions assayed as compared with the corresponding matched groups of animals treated with vehicle alone. Therefore, CB_1_ blockade did not affect *Cnr1* gene expression in PrAT. The CB_1_ protein levels in PrAT samples were analyzed by western blotting. The increase in *Cnr1* gene expression observed in obesogenic conditions was not followed by an increase in protein levels, which likely indicates a fast recycling rate. Densitometric analysis revealed that the expression levels of CB_1_ protein were not affected by AM251 treatment in any of the diet conditions assayed (data not shown).

### Effect of Obesogenic Diets and AM251 on the LXRα Expression in PrAT

**Figure 3:**
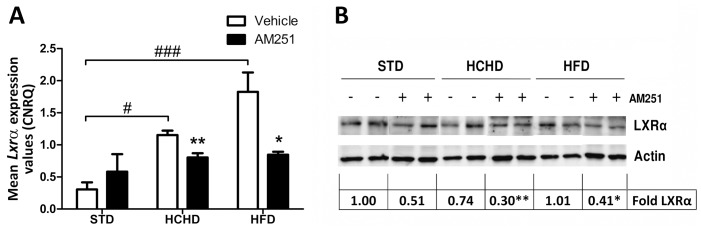
Effect of obesogenic diets and AM251 on the expression of the *Lxrα* transcription factor in PrAT. The *Lxrα* gene expression was determined in PrAT of STD, HCHD or HFD-fed animals after 14-day exposure to vehicle or AM251 (3 mg/kg, daily, i.p.). Columns are CNRQ means ± SEM (n = 8 animals per group). Differences between diets were analyzed by one-way ANOVA and Bonferroni’s *post-hoc* test, and comparisons between groups treated with AM251 or vehicle were analyzed by Student’s *t* test. #*p*<0.05 and ###*p*<0.001 denote significant differences compared with the STD-fed group treated with vehicle. *p<0.05 and **p<0.01 denote significant differences compared with the corresponding vehicle-treated group (**A**). Western blot analysis of the LXRα protein was determined in the three diet conditions treated with vehicle alone or AM251 (n = 6 animals per group). Two samples of each group are shown as representative. Relative LXRα protein levels were determined by densitometry corrected for beta-actin. Levels of LXRα are expressed as the fold over the STD-vehicle treated group (set as 1) as indicated at the bottom of the blot. *p<0.05 and **p<0.01 denote significant differences compared with the corresponding vehicle-treated group (**B**).

CB_1_ activation is implicated in lipogenesis [17], [19]. Thus, since LXRα is the main transcription factor that controls the expression of lipogenic enzymes [6], we sought to evaluate the impact of CB_1_ activity on the expression of this factor in different nutritional conditions. The *Lxrα* gene expression was evaluated in PrAT of rats fed obesogenic and normal chow diets. As shown in Figure 3A, the *Lxrα* expression was significantly higher in PrAT from rats fed either HCHD (*p*<0.05) or HFD (*p*<0.001) as compared with rats fed regular chow. Administration of AM251 significantly reduced the *Lxrα* expression in HCHD-fed (*p*<0.01) and HFD-fed rats (*p*<0.05) compared to their respective group rats receiving vehicle. This indicated that the high *Lxrα* expression levels found in rats fed obesogenic diets were, at least in part, dependent on the CB_1_-mediated response.

The western blot analysis revealed that LXRα protein expression did not appear to parallel the gene expression levels observed in the different groups of diets and vehicle-treated rats (Fig. 3B) However, LXRα levels were 0.30±0.07-fold (*p*<0.01) in the HCHD and 0.41±0.10-fold (*p*<0.05) in the HFD rats treated with AM251 (Fig. 3B), thus reinforcing the notion that LXRα expression is under the control of the CB_1_-mediated response, at least in PrAT of obese rats.

### Effect of Obesogenic Diets and AM251 on the *Srebf-1* and *ChREBP* Expression in PrAT

**Figure 4:**
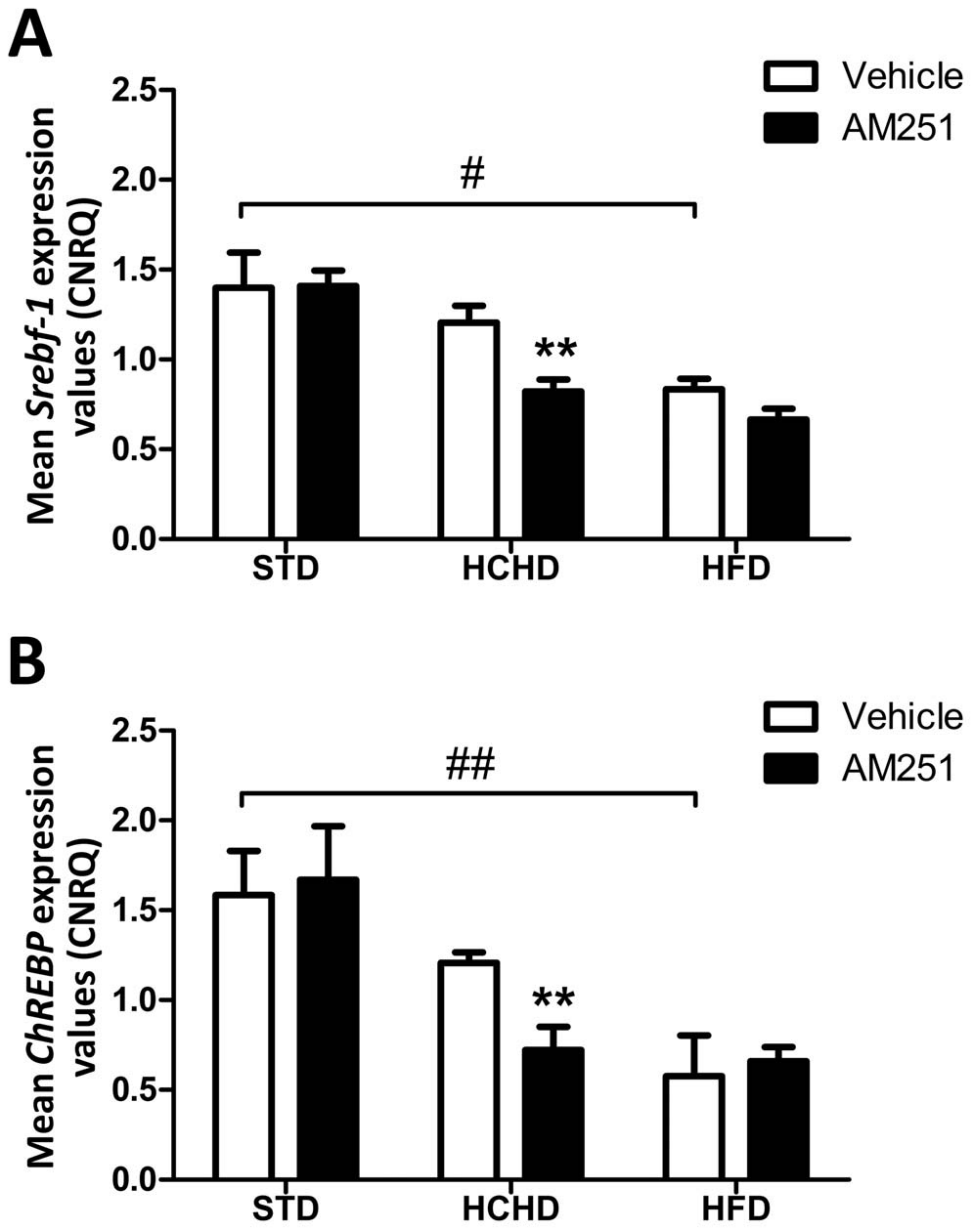
Effect of obesogenic diets and AM251 on the *Srebf-1* and *ChREBP* gene expression in PrAT. The gene expression of *Srebf-1* (**A**), and *ChREBP* (**B**), was determined in PrAT of STD, HCHD or HFD-fed animals after 14-day exposure to vehicle or AM251 (3 mg/kg, daily, i.p.). Columns are CNRQ means ± SEM (n = 8 animals per group). Differences between diets were analyzed by one-way ANOVA and Bonferroni’s *post-hoc* test, and comparisons between groups treated with AM251 or vehicle were analyzed by Student’s *t* test. #*p*<0.05, ##*p*<0.01 denote significant differences compared with the STD-fed group vehicle-treated. **p<0.01 denote significant differences compared with the corresponding vehicle-treated group.

As LXRα is a transcription factor for the *Srebf-1* and *ChREBP* genes [6], [31], [42], we then analyzed their gene expression levels in the different diet groups. As shown in Figure 4A and B, we found that the expression of *Srebf-1* and *ChREBP* detected in samples from the HCHD-fed group did not change significantly in comparison with the STD group. In contrast, a HFD promoted a significant decrease in the expression of *Srebf-1* (*p*<0.05) and *ChREBP* (*p*<0.01) (Fig. 4A, B). Treatment with AM251 in both the STD and the HFD groups did not affect significantly the *Srebf-1* or *ChREBP* expression levels compared with their respective groups receiving vehicle alone (Fig. 4A, B). Conversely, the expression levels of *Srebf-1* and *ChREBP* decreased significantly (*p*<0.01) under treatment with AM251 in samples from HCHD-fed rats. This observation suggests that CB_1_-mediated signalling contributes to sustain the *Srebf-1* and *ChREBP* gene expression in response to a diet rich in carbohydrates.

### Effect of Obesogenic Diets and AM251 on the Expression of Lipogenic Enzymes in PrAT

**Figure 5:**
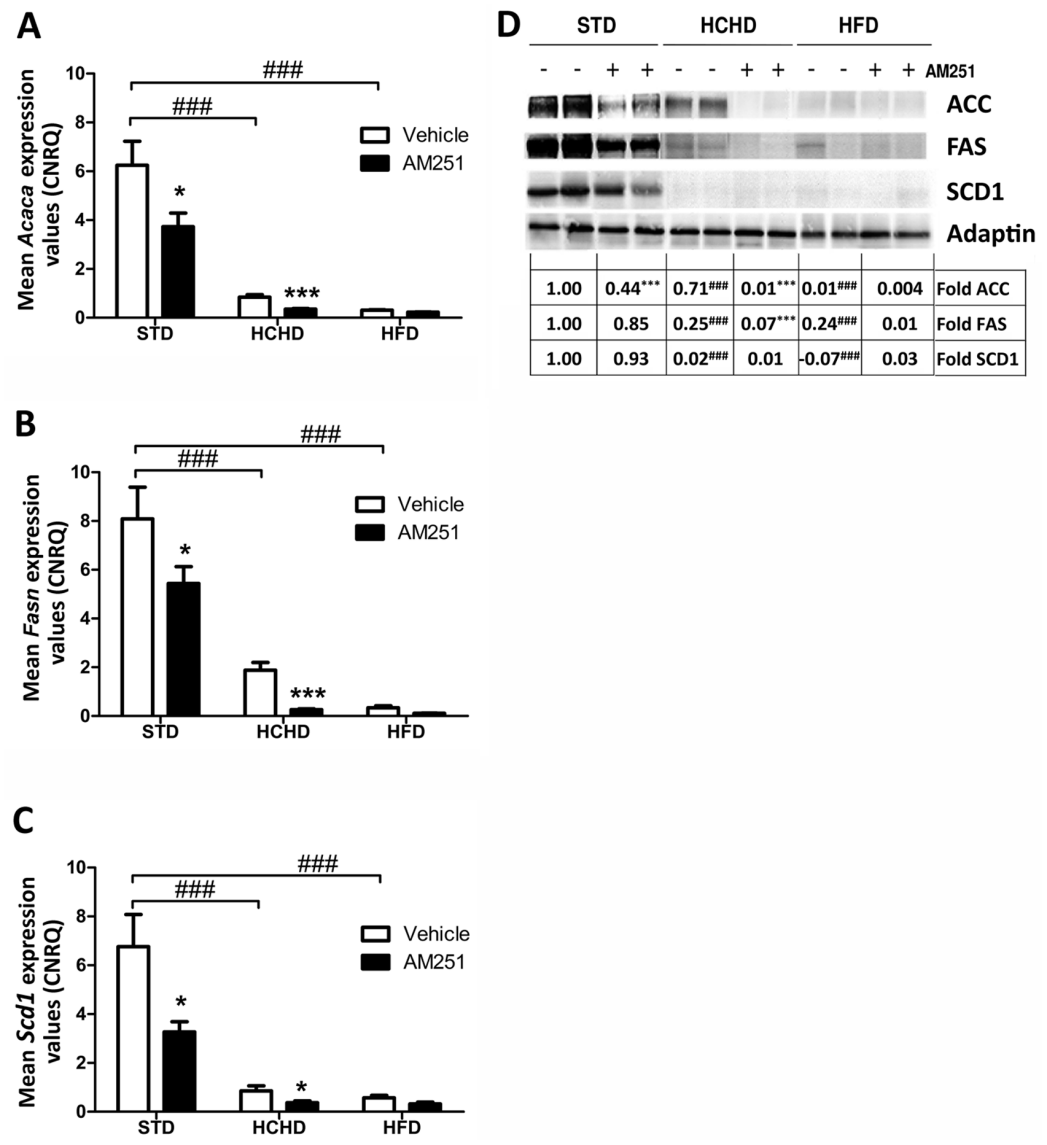
Effect of obesogenic diets and AM251 on the lipogenic enzyme gene and protein levels in PrAT. The gene expression of *Acaca* (**A**), *Fasn* (**B**) and *Scd1* (**C**) was determined after qPCR analysis in PrAT of STD, HCHD or HFD-fed animals vehicle-treated or AM251-treated (3 mg/kg, daily, i.p.). Columns are means ± SEM (n = 6 animals per group). Western blot analysis of protein content of ACC, FAS and SCD1 was determined in PrAT extracts (**D**). Two samples of each group are shown as representative. Relative ACC, FAS and SCD1 protein levels were determined by densitometry corrected for Actin. Protein levels are expressed as the fold over the STD-vehicle treated group (set as 1) for each protein as indicated at the bottom of the respective blot. Differences between diets were analyzed by one-way ANOVA and Bonferroni’s *post-hoc* test, and comparisons between groups treated with AM251 or vehicle were analyzed by Student’s *t* test. ###*p*<0.001 denotes significant differences compared with the STD-fed group treated with vehicle. *p<0.05 and ***p<0.001 denote significant differences compared with the corresponding vehicle-treated group.

We next analyzed the involvement of CB_1_ in the gene and protein expression of the lipogenic enzymes ACC, FAS and SCD1 in PrAT of rats fed the two obesogenic diets and a regular chow diet. We found that the gene expression levels of these enzymes (*Acaca*, *Fasn*, and *Scd1*) were dramatically down-regulated (*p*<0.001) in PrAT of animals fed HCHD and HFD as compared with the animals fed regular chow, though in the HCHD condition a remaining gene expression level of these enzymes was still measurable by qPCR (Fig. 5A–C). In samples from STD-fed rats, the treatment with AM251 slightly decreased (*p*<0.05) the expression levels of *Acaca*, *Fasn* and *Scd1* (Fig. 5A–C, respectively). Likewise, the AM251 treatment further reduced the expression levels of *Acaca* (*p*<0.001), *Fasn* (*p*<0.001) and *Scd1* (*p*<0.05) (Fig. 5A–C, respectively) to undetectable levels in obese rats fed a HCHD. The initial gene expression of these enzymes in PrAT of HFD-fed animals was so low that the inhibitory effects of AM251 could hardly be quantified by qPCR (Fig. 5A–C, respectively).

The western blot analysis revealed a remarkable reduction in protein levels of ACC, FAS (*p<0,001*) in PrAT of obese rats fed a HCHD or HFD and vehicle-treated, as compared with the levels found in samples from STD-fed rats (Fig. 5D). Also, SCD1 protein levels were drastically reduced in both HCHD and HFD conditions. Notably, the reduction in these protein levels was particularly dramatic in HFD conditions (Fig. 5D). Densitometric assessment of the ACC, FAS and SCD1 bands determined that treatment with AM251 significantly reduced the expression of ACC (*p*<0.001) in the STD group, while no significant changes were detected in FAS and SCD1 protein expression as compared with their matched vehicle-treated control groups (Fig. 5D). In the HCHD-fed group, the AM251 potentiated the reduction in protein levels of ACC (*p*<0.001) and FAS (*p*<0.001) to non-detectable levels (Fig. 5D), thus indicating that the expression levels of these lipogenic enzymes are dependent on CB_1_ activity in this diet condition. The meagre amounts of ACC, FAS and SCD1 proteins detected in PrAT of HFD-fed rats hardly allowed measuring the effects of AM251 (Fig. 5D).

### Effect of AM251 on CB_1_ Mediated Signalling in PrAT of Rats fed Obesogenic Diets for a Long Time

**Figure 6:**
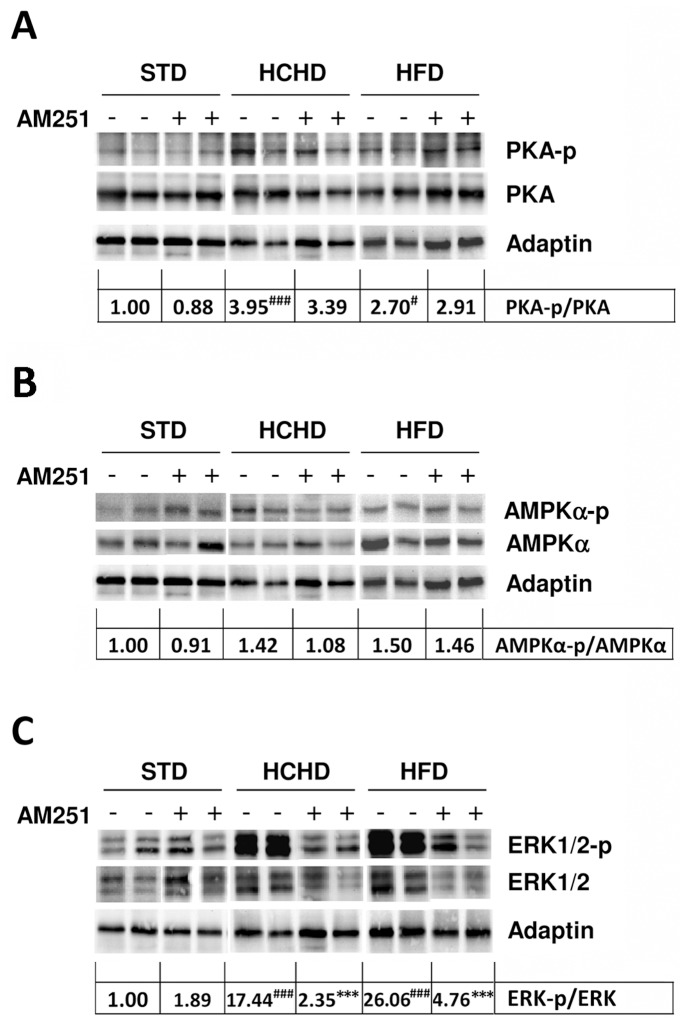
Effect of obesogenic diets and AM251 on PKA, AMPK and ERK phosphorylation status in PrAT. Western blot analysis of the phosphorylation status of the PKA enzyme (**A**) AMPK (**B**) and ERK1/2 (**C**) was determined in PrAT of STD, HCHD or HFD-fed animals treated with vehicle alone or AM251. Adaptin is shown as equal protein loading control. The ratio of densitometric values of the PKA-p/PKA, AMPK-p/AMPK and ERK-p/ERK bands are shown at the bottom of the respective blots. Differences between diets were analyzed by one-way ANOVA and Bonferroni’s *post-hoc* test, and comparisons between groups treated with AM251 or vehicle were analyzed by Student’s *t* test. #*p*<0.05 and ###*p*<0.001 denote significant differences compared with the STD-fed group treated with vehicle (set as 1). ***p<0.001 denotes significant differences compared with the corresponding vehicle-treated group.

The above results suggested that CB_1_-mediated signalling regulates the expression of lipogenic enzymes, probably through modulating the activity or the expression of their transcription factors. Moreover, this modulation would depend on the context of the diet. The activity of LXRα is negatively regulated by phosphorylation, which depends in part on the activation of the cAMP/PKA/AMPK pathway [27], [30]–[32]. It is stated that CB_1_ agonists inhibit the PKA/AMPK pathway through coupling the Gi/o protein. Consequently, we next sought to investigate the phosphorylation (activation state) of the kinases PKA and AMPK in PrAT from animals fed the different diets and treated with vehicle or AM251. The PKA-p/PKA ratio was analyzed by western blot. This analysis showed that no significant changes were detected in the levels of PKA between samples of the different diet groups, either vehicle-treated or AM251-treated (Fig. 6A). Indeed, densitometric evaluation of the PKA-total protein content normalized against Adaptin indicated no variations between samples of all groups (data not shown). The presence of a phosphorylated PKA (PKA-p) band was evident in all PrAT samples (Fig. 6A). In contrast to what was expected, densitometric analysis showed that the PKA-p band was enhanced 3.95-fold (p<0.01) in the HCHD group and 2.7-fold (p<0.05) in the HFD group, compared to the basal levels found in vehicle-treated STD samples (Fig. 6A). The CB_1_ blockade by AM251 in any of the three diet conditions was expected to increase the PKA-p status; however no significant modification in its phosphorylation levels was observed, as compared with their respective matched vehicle-treated diet groups (Fig. 6A).

The AMPK-p/AMPK ratio was also examined in the same blot for PKA-p/PKA (Fig. 6B). In our assay conditions, no significant changes were observed in AMPK content when normalized against Adaptin by densitometry (data not shown). The antibody used in this study to detect the activated AMPK form recognizes the Thr172 phosphorylated residue which is phosphorylated by the LKB1 protein that, in turn, is a substrate for PKA. The analysis by western blot showed no significant variations in AMPK-p status in any of the three diet conditions in vehicle-treated or AM251-treated rats (Fig. 6B) and the densitometric analysis of the AMPK-p/AMPK ratio confirmed this observation (Fig. 6B).

As ERK1/2 (p42/p44 MAPK) is directly involved in G_i/o_-coupled CB_1_ receptor-mediated signalling [35], we also evaluated the phosphorylation status of ERK1/2 (ERK1/2-p) in PrAT samples. The ERK1/2 and ERK1/2-p protein forms were analyzed by western blot (Fig. 6C). Assessment of the ERK1/2 total protein content normalized against Adaptin showed no significant variations between samples from all groups (Fig. 6C). However, the phosphorylation status of ERK1/2 showed dramatic variations depending on the group samples (Fig. 6C). Thus, densitometric analysis revealed that the ERK1/2-p/ERK ratio was significantly enhanced 17.44-fold (p<0.001) in PrAT from HCHD animals and 26.06-fold (p<0.001) in HFD animals over the basal levels found in the vehicle-treated STD group (Fig. 6C). The extent of the ERK1/2-p dependence on CB1 activity was analyzed in samples from animals treated with AM251. While no major differences were found between the vehicle-treated and AM251-treated STD-fed rats, protein analysis showed a dramatic decrease in the ERK-p/ERK ratio (*p*<0.001) in those samples from rats fed obesogenic diets and treated with AM251. Therefore, this last finding indicates that the ERK-p in PrAT of obese animals is dependent on CB_1_-mediated signalling. Moreover, even though the PKA pathway appeared activated, the latter result clearly indicates that the CB_1_ signal remains operational in the PrAT of diet-induced obese animals, but it is biased towards the ERK-signalling pathway.

## Discussion

CB_1_-mediated signalling is involved in the enhancement of the lipogenesis pathway in the liver [24], [27]. However, the contribution of CB_1_ to lipogenesis in different white adipose depots is not well studied. It is argued that increased circulating levels of endocannabinoids and CB_1_ hyperactivity contribute significantly to increased fat deposits, the hallmark of obesity [19], [24], [26], [43], [44]. Accordingly, the CB_1_ inverse agonist AM251 reduces body weight in rats fed a diet inducing obesity, and this effect appears to be related to a reduction in food intake [23], [41]. We used a similar *in vivo* model to study the impact of CB_1_ blockade in the expression of lipogenic genes in PrAT of rats fed two types of hypercaloric diets. We confirmed that in our experimental conditions the CB_1_ blockade by AM251 indeed reduced body weight in obese rats fed a HCHD or a HFD.

The data shown here demonstrate that *Cnr1* gene expression is up-regulated in PrAT after a long period of HCHD or HFD feeding. This is in contradiction with other results found in human samples where *Cnr1* mRNA levels are decreased in the visceral adipose tissue of obese individuals [19]. This discrepancy could be attributable to differences between human and rat endocannabinoid physiological functions or most probably to the source of the adipose tissue. In our case we used perirenal fat which is part of the retroperitoneal mass of the white adipose tissue. Indeed, the high *Cnr1* gene expression might be a response to chronic circulating endocannabinoids that promote continuous internalization and degradation of the CB_1_ receptor, as has been extensively described in both humans and animal models [43]–[46]. Nevertheless, our data suggest that the CB1 receptor does not regulate its own gene expression.

Here we show that *Lxrα* gene expression is up-regulated in PrAT of rats fed obesogenic diets. As obesity can be related to inflammation in adipose tissue and macrophages express high levels of LXRα [47], [48], we considered the possibility that a high presence of macrophages could account for the *Lxrα* up-regulation. However, histological analysis from all groups showed no inflammation in the PrAT and specific anti-CD68 macrophage immunostaining proved negative in all samples (data not shown). Also, it is important to note that macrophages migration is described to be induced mainly in mesenteric adipose tissue in obese mice [49]. Therefore, we considered that the increase in *Lxrα* gene expression comes mainly from adipocytes in the PrAT. CB_1_ blockade induced the down-regulation in the expression of the gene and protein LXRα, thus indicating that this expression is under the control of CB_1_.

We found that the *Srebf-1* and *ChREBP* gene expression was decreased in PrAT of animals fed a HFD. Since these genes are targets for LXRα, this observation could reflect a failing in the functionality of the LXRα transcription factor inherent to the HFD condition. The fact that treatment with AM251 did not further down-regulate these low levels of *Srebf-1* and *ChREBP* suggests that CB_1_ is not functional in PrAT of HFD-fed rats. In the HCHD groups we did not observe the same effect on these genes. Indeed, the expression in *Srebp-1* and *ChREBP* genes remained at levels found in the STD group. Nevertheless, AM251 treatment induced a significant decrease in their expression, thus suggesting that in this nutritional condition the expression of *Srebf-1* and *ChREBP* factors is indeed regulated by CB_1_-mediated signalling.

These observations are in consonance with the lipogenic enzyme levels found in the obesogenic diet conditions. The AM251 treatment in regular chow-fed animals slightly down-regulated the *Acaca, Fasn* and *Scd1* gene expression, indicating that CB_1_-mediated signalling contributes in controlling their gene expression in PrAT. Our *in vivo* assays consisted of carbohydrate or fat over-feeding for a long time, and in these conditions we observed a drastic down-regulation of *Acaca, Fasn* and *Scd1* gene expression in PrAT. Our results are in line with recent data showing the lowered mRNA levels of *Acaca* and *Fasn* in the adipose tissue of human obese subjects as compared with samples from lean subjects [9], [10], [50]. Nevertheless, our findings further point to some differences depending on the type of obesogenic diet. Thus, while in the HFD nutritional condition the expression of these enzymes is completely ablated, the PrAT samples of the HCHD group still showed a remnant in the gene expression of these enzymes. Moreover, these levels are dependent on CB_1_ since the AM251 treatment promoted the total down-regulation of *Acaca, Fasn* and *Scd1 e*xpression.

Our results indicate that the PKA-p status in PrAT of obese animals is no longer controlled by CB_1_ signalling since the PKA is highly phosphorylated and AM251 treatment did not affect it. A previous report examining the levels of gene expression in adipose tissue from obese mice revealed a decrease in G_i/o_ expression associated with obesity [51] which could account for a permanent PKA-p state. The increase in PKA-p status that we observed in PrAT of obese rats likely has a negative repercussion on the LXRα transcriptional activity that, in turn, would negatively affect the *Srebf-1* and *ChREBP* gene expression levels. Indeed, we observed a reduced expression of these genes, at least in HFD-fed rats. Accordingly, the presence of lipogenic enzymes is dramatically reduced in terms of gene and protein expression levels in PrAT of HFD-fed rats. In HCHD conditions, a high PKA-p status was also observed in PrAT; however, the *Srebf-1* and *ChREBP* gene expression remained as in normal chow fed rats. Nevertheless, the reduction in the expression of their target genes *Acaca, Fasn y Scd1* suggests that the activity of the protein forms of SREBP and ChREBP is probably diminished. Interestingly, CB1 blockade down-regulated the gene expression of *Lxrα, Srebf-1* and *ChREBP* factors in rats fed a HCHD, and consequently the already lowered expression of lipogenic enzymes was completely abolished. This last observation indicates that the gene expression of these factors and their target genes is still CB_1_-dependent in the HCHD condition.

AMPK activation is reported to phosphorylate LXRα, thereby repressing expression of SREBP [31]. As a result, expression of the target lipogenic enzyme genes is, in turn, suppressed [27], [30], [32]. The antibody used in this study to detect AMPK-p recognizes the Thr172 phosphorylated residue which is phosphorylated via the PKA pathway. However, we failed to demonstrate changes in this AMPK-p status in all the nutritional conditions, either vehicle-treated or AM251-treated groups, and indeed lean and obese rats showed equal basal levels of this AMPK-p form. Further analysis, including different antibodies recognizing other phosphorylated residues in AMPK and additional *in vivo* experiments for collecting samples over a time course will be addressed in order to elucidate this question.

Analysis of the activation state of the kinase ERK, which is involved in CB_1_-mediated signalling, also revealed a high ERK-p status in PrAT of obese animals. Our results demonstrating that AM251 drastically prevents this ERK phosphorylation point out that CB_1_ is indeed operational in PrAT of obese animals. Thus, our data indicate a simultaneous PKA-p and ERK-p status in PrAT of animals fed obesogenic diets as opposed to animals fed a normal chow diet. Since ERK-p is in turn involved in the activation of SREBP by phosphorylation [37], [52], [53] these observations reflect a contradictory signal triggered in PrAT of animals exposed to diet-induced obesity for a long time; i.e., suppression and activation of lipogenic gene expression simultaneously, with the ERK-p status being CB_1_ dependent.

Overall, our findings suggest that, after a long period with a high-calorie diet, the CB_1_-mediated signalling pathway that specifically induces activation of lipogenesis is seriously compromised without affecting other signalling pathways such as the ERK1/2 pathway, which is highly activated in PrAT of obese rats. The specific role of ERK in the CB_1_-mediated signalling pathway in obese conditions needs further investigation. In this regard, some authors have reported the implication of ERK1/2 in visceral obesity through promoting SREBP phosphorylation and indeed, inhibition of such phosphorylation protects mice from fatty liver and visceral obesity [37]. On the other hand, activation of ERK pathway appears to be involved in adipocyte lipolysis by phosphorylating the hormone-sensitive lipase (HSL) and increasing the activity of HSL [54]. Further research is needed to understand how ERK1/2 pathway might affect perirenal fat depot and its influence on surrounding tissues including liver and kidney.

Finally, the observation that obese rats maintained on a HCHD still show a remnant lipogenic gene expression in PrAT, and that this is CB_1_-dependent, could be one of the causes contributing to hindering the treatment of obesity induced by the abusive intake of carbohydrates. Therefore, in such cases, a low carbohydrate diet combined with CB_1_-receptor blockade would be advantageous for the treatment of HCHD-induced abdominal obesity.
